# Compact and cGMP-compliant automated synthesis of [^18^F]FSPG on the Trasis AllinOne™

**DOI:** 10.1186/s41181-024-00322-7

**Published:** 2025-01-17

**Authors:** Rizwan Farooq, Thibault Gendron, Richard S. Edwards, Timothy H. Witney

**Affiliations:** 1 School of Biomedical Engineering & Imaging Sciences, King’s College London, St Thomas’ Hospital, London SE1 7EH, UK; 2 GIGA-CRC Human Imaging, Cyclotron Research Centre, University of Liege, Liege, Belgium

**Keywords:** [^18^F]FSPG, Trasis, Automation, Positron emission tomography, SPE purification

## Abstract

**Background:**

(*S*)-4-(3-^18^F-Fluoropropyl)-L-glutamic acid ([^18^F]FSPG) is a positron emission tomography radiotracer used to image system x_C_^−^, an antiporter that is upregulated in several cancers. Not only does imaging system x_C_^−^ with [^18^F]FSPG identify tumours, but it can also provide an early readout of response and resistance to therapy. Unfortunately, the clinical production of [^18^F]FSPG has been hampered by a lack of robust, cGMP-compliant methods. Here, we report the automated synthesis of [^18^F]FSPG on the Trasis AllinOne™, overcoming previous limitations to provide a user-friendly method ready for clinical adoption.

**Results:**

The optimised method provided [^18^F]FSPG in 33.5 ± 4.9% radiochemical yield in just 35 min when starting with 18–25 GBq. Importantly, this method could be scaled up to > 100 GBq starting activity with only a modest reduction in radiochemical yield, providing [^18^F]FSPG with a molar activity of 372 ± 65 GBq/µmol and excellent radiochemical purity (96.8 ± 1.1%). The formulated product was stable when produced with these high starting activities.

**Conclusions:**

We have developed the first automated synthesis of [^18^F]FSPG on the Trasis AllinOne™. The method produces [^18^F]FSPG with excellent radiochemical purity and in high amounts suitable for large clinical trials and off-site distribution. The method expands the number of synthesis modules capable of producing [^18^F] FSPG and has been carefully designed for cGMP compliance to simplify regulatory approval for clinical production. The methods developed for the purification of high-activity [^18^F]FSPG are transferrable and should aid the development of clinical [^18^F]FSPG productions on other synthesis modules.

## Background

Metabolic reprogramming in cancer is required to meet the unique demands of transformed cells (Luengo et al. 2017). This aberrant metabolism can be visualised by radiotracers that target these perturbed metabolic processes with positron emission tomography (PET) imaging (Aizhi et al. 2011). The detection and staging of tumors using [^18^F]2-fluoro-2-deoxy-D-glucose ([^18^F]FDG) PET, which relies on the increased glucose uptake by these tumours, demonstrates the clinical value of such radiotracers (Mankoff et al. 2007). Radiotracers targeting increased amino acid transporter expression can also be used for tumour detection, in some cases improving tumour contrast compared to [^18^F]FDG, or providing additional prognostic information (Pantel et al. 2018; Huang and McConathy 2013; McConathy and Goodman 2008).

(*S*)-4-(3-^18^F-Fluoropropyl)-L-glutamic acid ([^18^F]FSPG) is a fluorine-18 labelled glutamate analogue which specifically targets the cystine/glutamate antiporter (system x_c_^−^) (Koglin et al. 2011). System x_c_^−^ is highly expressed in several cancer types whilst its expression in healthy tissues is low (Koglin et al. 2011). PET imaging with [^18^F]FSPG, therefore, leads to high tumor-to-background ratios and favourable dosimetry, as has been demonstrated clinically in head and neck squamous cell cancer, non-small cell lung cancer, hepatocellular carcinoma, prostate cancer, and brain tumors (Sharkey et al. 2024; Wardak et al. 2022; Park et al. 2020; Baek et al. 2012; Mittra et al. 2016; Kavanaugh et al. 2016; Cheng et al. 2019).

The biological role of system x_c_^−^ is to transport extracellular cystine into the cell, a process facilitated by its exchange for intracellular glutamate with a 1:1 stoichiometry (Bannai and Ishii 1988). Cystine is the dimeric form of cysteine, which is the rate-determining precursor for glutathione [GSH) production (Lu 2009). GSH is essential for modulating cellular redox and, through its upregulation, protects cancer cells from both endogenous and therapy-induced oxidative stress (Bansal and Simon 2018; Liu et al. 2020).

The link between system x_c_^−^ and the cancer cell’s ability to maintain cellular redox homeostasis places system x_c_^−^ as one of the key gatekeepers of therapy resistance, and consequently, has been explored as a therapeutic target (Liu et al. 2020). Concurrently, radiotracers targeting system x_c_^−^ have become important tools to detect early response to therapy (Greenwood et al. 2019, 2023; McCormick et al. 2019; Sambasivan et al. 2024), as well as to visualise target-engagement of inhibitors that bind to this metabolic vulnerability (Greenwood et al. 2022; Smith et al. 2023). [^18^F]FSPG has also been used to image other diseases where system x_c_^−^ expression is altered, or cellular redox processes are perturbed – including multiple sclerosis, cerebral ischemia, inflammatory bowel disease, and argininosuccinic aciduria (Martín et al. 2016; Domercq et al. 2016; Seo et al. 2022; Gurung et al. 2024).

As interest in preclinical and clinical research using [^18^F]FSPG continues to grow, so does the demand for its reliable and widely available production. Several automated syntheses have been reported for [^18^F]FSPG, and recent technological advances have established clinical production processes (Edwards et al. 2021; Brown et al. 2023; Lin et al. 2023). However, the use of bespoke consumables and external components in these methods is unattractive for widespread dissemination and regulatory compliance. Furthermore, to maximise the number of sites capable of producing [^18^F]FSPG, there is a requirement to establish robust, cGMP-compliant syntheses on multiple mainstream synthesis modules.

Here, we report the first automated synthesis of [^18^F]FSPG on the widely used Trasis AllinOne™ (Trasis AIO™). A compact cassette design contained consumables and reagents within the cassette, removing undesirable external components. The process reliably produced [^18^F]FSPG at high radioactivity levels suitable for clinical use. Furthermore, the commercial availability of all the required components will facilitate this method’s widespread adoption and clinical approval at other production sites.

## Materials and methods

### General

**Fig. 1:**
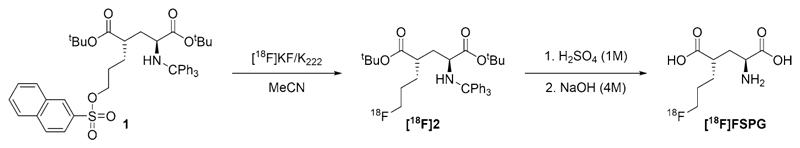
Synthetic scheme for [^18^F]FSPG production

A CRC-55tR dose calibrator (Capintec Inc.) was used to measure radioactivity. The automated synthesis module used in the study was the Trasis AIO™ (Trasis SA), and the software version 2.33 was used for sequence development and synthesis runs. All experiments were performed in a Comecer hot cell. The consumables for the Trasis AIO™ were purchased from Trasis SA, including the Trasis AIO™ starter set for R&D (Part #:7730), and 6 mL type-1 glass reactors (Part #:7613). The [^18^F]FSPG precursor (**1**, Fig. 1, Product #: 3193) and non-radioactive reference (Product #: 3194) were purchased from ABX GmbH, Radeberg, Germany. [^18^F]Fluoride was produced using a GE PET Trace 880 Cyclotron. ^18^O water (98%) was purchased from Rotem Industries Limited. Solid phase extraction (SPE) cartridges were purchased from Waters, including QMA Carbonate 46 mg sorbent (Part #: 186,004,540), mixed cation exchange (MCX) Oasis (Part #:186,003,516) and Alumina N Plus Long cartridges (Part #: WAT020510). ENVI-Carb (1.0 g, 12 mL) cartridges were purchased from Sigma Aldrich-Merck. ENVI-Carb cartridges were connected to the Trasis tubing using an adaptor cap for SPE tubes (AH0-7191, Phenomenex). Alumina N Long Cartridges were conditioned with sterile water (10 mL) followed by air (10 mL) before use. ENVI-Carb cartridges were conditioned with ethanol (10 mL) and phosphate buffered saline (PBS, 10 mL) followed by air (10 mL) before use. European Pharmacopeia Grade PBS was purchased from Fisher Scientific (Part #:12,990,704). *o*-Phthalaldehyde (OPA) reagent (Part #: 5061–3335) was purchased from Agilent Technologies. Kryptofix 222/carbonate solution was prepared in house [Kryptofix 222 (Fisher Scientific, 10,523,094; 8.0 mg, 21.2 μmol), potassium carbonate (Sigma Aldrich-Merck, 590,681; 1.1 mg, 8.0 μmol), acetonitrile (0.65 mL), and water (0.20 mL)].

### Cassette design

**Fig. 2:**
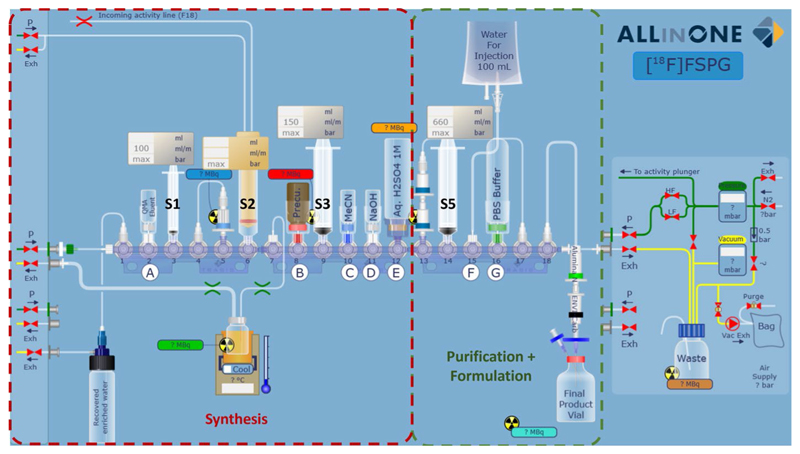
Schematic of [^18^F]FSPG cassette design. Cassette positions are labelled 1–18. Reagents are labelled A-G (See Table 1). Syringes are labelled S1-S5 (syringe driver 4 is unused). Red or green dashed lines refer to the sections of the cassette predominantly used for [^18^F]FSPG synthesis or purification, respectively

The cassette designed for the automated synthesis of [^18^F]FSPG prioritised cGMP compliance and ease of clinical translation. All reagents were contained within the mounted cassette to avoid needing external reagents and to provide the user a ‘plug-and-play’ experience. Cartridges were positioned to align with the Trasis AIO™’s activity detectors so the procedure could be followed in real-time. The cassette design also looked to maximise the number of Trasis AIO™ modules that would be compatible with the sequence. The cassette was, therefore, limited to 3 manifolds to facilitate synthesis on all Trasis AIO™ configurations (from 18 to 36 valves). Furthermore, syringe driver 4 (Fig. 2) was avoided, as it is not available for all AIO configurations. Finally, to maximise final product purity, the components required for [^18^F]FSPG purification and formulation (Fig. 2, green dashed lines) were separated from the necessary components for the multi-step synthesis (Fig. 2, red dashed lines).

### Set-up

**Fig. 3:**
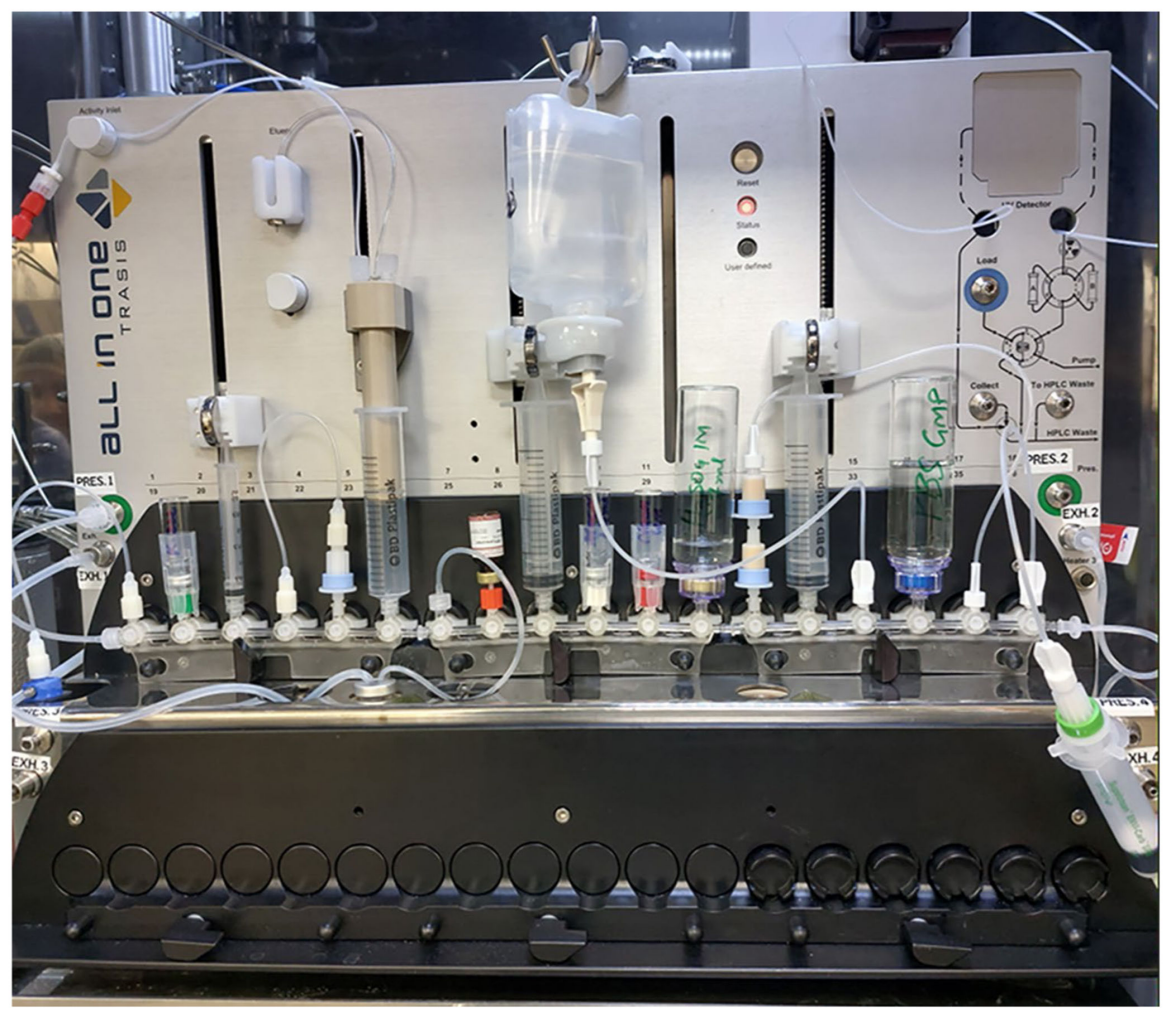
Photo of mounted cassette after loading the reagents

The cassette was assembled without adding the reagents according to Fig. 2 and Table 1. Photos of the assembly are provided in Supplementary Fig. 1. Once the machine test was completed, the cassette was mounted, and the cassette test was performed. Finally, the reagents were placed in their correct positions according to Table 1 (Fig. 3).

### Preliminary steps: precursor solubilisation and cartridge conditioning

Before delivery of radioactivity to the Trasis AIO™ from the cyclotron, automated pre-cursor solubilisation and cartridge conditioning were performed. The precursor was automatically solubilised by transferring the MeCN from CP10 to CP08 using pressure/vacuum. Subsequently, Syringe 3 was used to condition the MCX cartridges with PBS (20 mL), followed by water (20 mL) at 20 mL/min. The cartridges were then flushed with N_2_ (1000 mbar).

### Optimised automated synthesis of [^18^F]FSPG

[^18^F]Fluoride was produced by a GE PET Trace 880 cyclotron (GE Healthcare) by 16 MeV irradiation of 3.0 mL enriched [^18^O]H_2_O at the Positron Emission Radiopharmaceutical Laboratory (PERL), St. Thomas’ Hospital (London, UK). [^18^F]Fluoride was transferred via a Radionuclide Delivery System (RNDS) to the Trasis AIO™ synthesis module, where it was collected in Syringe 2 via the activity plunger (Figs. 2 and 3). The [^18^F]fluoride was then trapped on a QMA light SepPak cartridge (46 mg Sorbent), and the ^18^O water was eluted into the recovery vial. The trapped [^18^F]fluoride was eluted to the reactor with Kryptofix/carbonate solution (450 µL) using syringe 1. The [^18^F]fluoride/kryptofix/carbonate mixture was dried under a flow of N_2_ and reduced pressure at 110–125 °C for 11 min. The reactor was then cooled to 55 °C before the addition of [^18^F]FSPG precursor (6.0 mg; **1**, Fig. 1) in MeCN (1.2–1.4 mL). The reaction was heated for 5 min at 110 °C before the reactor temperature was reduced to 70 °C and H_2_SO_4_ (3.0 mL, 1 M) was added. The mixture was then heated at 105 °C for a further 4 min. The reactor was cooled to 70 °C before adding NaOH (1.9 mL, 4 M). The reactor was kept at 70 °C for 5 min before turning off the reactor heater. The reaction mixture was then transferred to a 50 mL vial containing H_2_SO_4_ (4.0 mL, 1 M) and mixed using Syringe 2. The crude was then passed through two Oasis MCX cartridges at 20 mL/min to extract the [^18^F]FSPG product before washing with water for injection (WFI) (20 mL) at 20 mL/min. Syringe 3 was then used to elute the [^18^F]FSPG product with PBS (10 mL) through the Alumina N Long Cartridge, the ENVI-Carb (1.0 g, 12 mL) SPE cartridge, and a sterile 0.22 µm filter into a sterile vial. Finally, nitrogen pressure was used to flush the remaining product to the product vial. Activity, pressure and temperature trending are represented in Supplementary Fig. 2. The final sequence is provided in the supplementary materials.

### Characterisation and quality control

Before HPLC analysis, precolumn derivatisation of [^18^F]FSPG with OPA was performed (Supplementary Fig. 3A) following a previously reported method (Edwards et al. 2021). Formulated [^18^F]FSPG (20 µL) was added to OPA reagent (20 µL) before dilution and mixing with PBS (80 µL). The reaction mixture was left for 5 min to allow full conversion of [^18^F]FSPG to its corresponding OPA adduct ([^18^F]FSPG-OPA) before injection.

Analytical RP-HPLC was performed on an Agilent 1200 HPLC system equipped with a 1200 Series Diode Array Detector and a GABI Star NaI(Tl) scintillation detector (energy window 400–700 keV; Raytest) or an Agilent 1260 HPLC system equipped with a 1260 Series Diode Array Detector and a Flow RAM detector (LabLogic). Radiochemical purity (RCP) and product identity were analysed using the following conditions: Column = Chromolith C18 (100 × 4.6 mm), Merck Millipore; solvent A = H_2_O (0.1% TFA), solvent B = MeOH (0.1% TFA); flow rate = 3 mL/min; UV detector = 314 nm; gradient = 0–10 min, 10–90% B; 10–15 min, 90% B. Molar activity (A_m_) was measured using the following conditions: Column = Chromolith C18 (100 × 4.6 mm), Merck Millipore; solvent A = H_2_O (0.1% TFA), solvent B = MeOH (0.1% TFA); flow rate = 3 mL/min; UV detector = 314 nm; gradient = 0–2 min, 5–30% B; 2–17 min, 30–50% B; 17–20 min, 50–95% B; 20–23 min, 95–5% B.

**Fig. 4:**
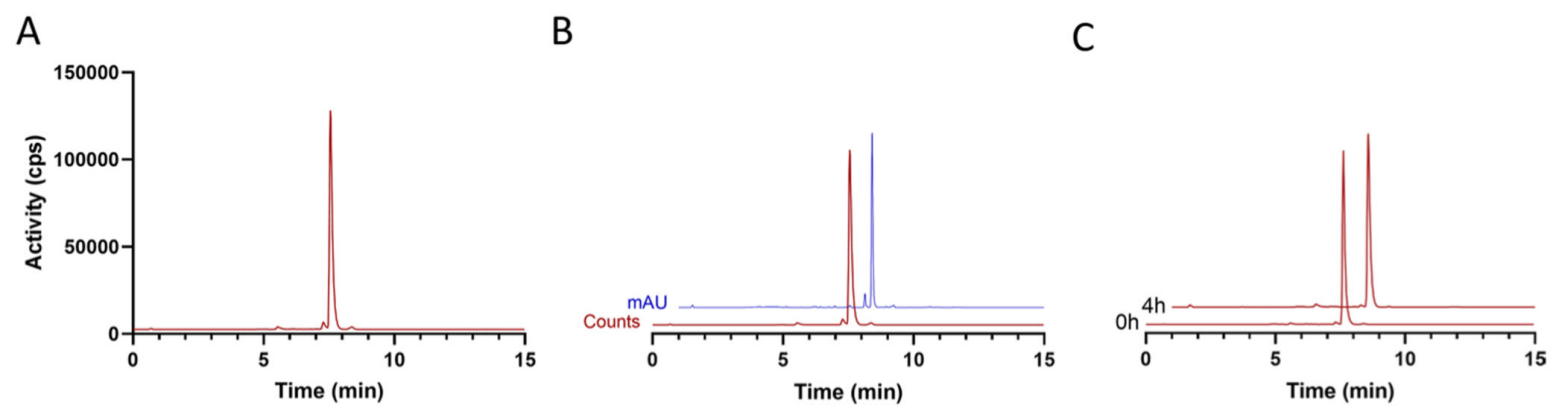
Representative HPLC chromatograms of [^18^F]FSPG after OPA derivatisation. **A** Radio-chromatogram of the final product. **B** Radio (red) and UV chromatogram (blue) of [^18^F]FSPG spiked with cold standard ([^19^F] FSPG). **C** [^18^F]FSPG formulation stability at room temperature

Molar activity (A_m_) was calculated using the following equation: A_m_ = Activity injected (GBq)/amount injected (µmol). The measured ‘amount injected (µmol)’ was calculated by calibrating the absorbance measured for [^19^F]FSPG-OPA at different concentrations (Supplementary Fig. 4). RCP was measured immediately and at 4 h post-synthesis to check formulation stability (samples were diluted to < 100 MBq/mL, if required, to ensure detector saturation did not occur). To confirm product identity by co-elution, OPA derivatisation was performed with the addition of the [^19^F]FSPG ‘cold’ reference (1.0 mg/mL in PBS, 20 µL) to the derivatisation reaction mixture (OPA, 20 µL), [^18^F] FSPG product (20 µL), [^19^F]FSPG reference (20 µL) and PBS (80 µL). Representative chromatograms for analysis of [^18^F]FSPG RCP, A_m_, product identity and formulation stability are shown in Fig. 4 and Supplementary Fig. 3B. Additionally, chromatograms for the formulation buffer and OPA are provided for reference in Supplementary Fig. 5. The pH of the final formulated product was measured using pH indicator strips (Product #: 10,642,751, Fisher Scientific). A summary of the QC results for individual batches is shown in Supplementary Table 1.

## Results

**Fig. 5:**
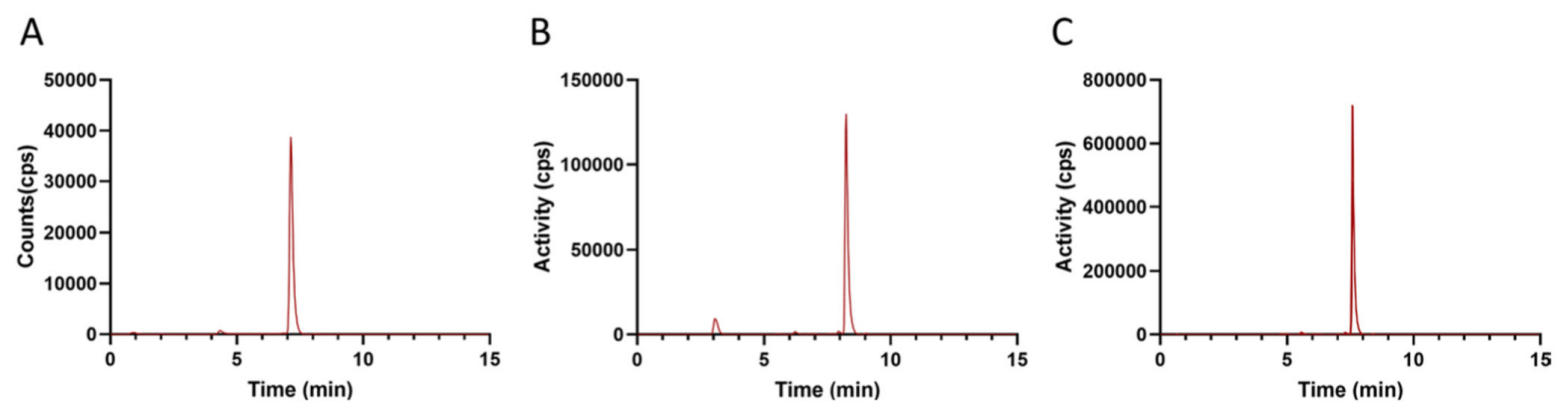
Representative radio-HPLC chromatograms of [^18^F]FSPG, showing the effect of SPE cartridge selection on final product purity. **A** [^18^F]FSPG produced with low starting activity (< 5 GBq) using a Hyper-Carb (500 mg) cartridge for purification. **B** [^18^F]FSPG produced with high starting activity (> 45 GBq) using a Hyper-Carb (500 mg) cartridge for purification. **C** [^18^F]FSPG produced with high starting activity (≤ 124 GBq) using an ENVI-Carb (1.0 g) cartridge for purification

Automated [^18^F]FSPG radiosynthesis on the Trasis AIO™ was based on our previously reported method using the GE Fastlab (Edwards et al. 2021). Building on this prior knowledge, we initially tested the automated synthesis with low starting activities (< 5 GBq), which produced [^18^F]FSPG in good radiochemical yield (RCY) and high RCP (34.6 ± 6.1% and 96.9 ± 0.7% respectively; Table 2, entry 1; Fig. 5A). However, when the starting activity was increased to > 45 GBq, the RCP dropped by ~ 3% to 94.0 ± 1.4% (Table 2, entry 2; Fig. 5B) and was further reduced to 93.1 ± 1.7% when over 90 GBq starting activity was used (Table 2, entry 3). Using a 2.0 g ENVI-Carb cartridge instead of the hypercarb cartridge led to an improved RCP (97.0 ± 0.1%) but, unfortunately, had a detrimental effect on RCY (Table 2, entry 4). The smaller 1.0 g ENVI-Carb cartridge was used next, which improved RCP and maintained a good RCY (Table 2, entries 5–7; Fig. 5C).

The final optimised process provided formulated [^18^F]FSPG with high RCP (> 96%) regardless of the starting activity in just 35 min. Good RCYs were observed for both medium (37–67 GBq) and higher scale (96–124 GBq) syntheses, producing [^18^F]FSPG in 26.2 ± 0.8% RCY and 25.3 ± 1.1% RCY, respectively. Interestingly, when performing the optimised synthesis with lower amounts of starting activity (18–25 GBq), the RCY increased to 33.5 ± 4.9%, suggesting that radiolysis may affect the RCY at higher activities. High activity productions (96–124 GBq) produced up to 23 GBq of [^18^F]FSPG formulated in PBS (final formulation, pH 7). Despite the high activity concentrations (up to 1.15 GBq/mL), the formulated product was stable with no drop in RCP 4 h post-synthesis (96.2 ± 0.6%; Fig. 4C). The molar activity of the final product was 372 ± 65 GBq/µmol.

## Discussion

Clinical and preclinical research using [^18^F]FSPG has expanded in recent years as its ability to image tumours and report on tumour redox status has been established (Sharkey et al. 2024; Park et al. 2020; Baek et al. 2012; Mittra et al. 2016; Kavanaugh et al. 2016; Greenwood et al. 2019, 2023; McCormick et al. 2019; Sambasivan et al. 2024). As the demand for [^18^F]FSPG increases, methods for its reliable production in amounts and quality suitable for clinical application are required. The reported synthesis has also been established to support a clinical study using [^18^F]FSPG at our Center (NCT05889312). While progress towards an ideal automated process for clinical production has been made recently, these methods still require external reagents and bespoke consumables for high-activity production of [^18^F]FSPG (Edwards et al. 2021; Lin et al. 2023). Furthermore, [^18^F]FSPG has yet to be automated on the widely used Trasis AIO™.

Conditions we previously reported to synthesise [^18^F]FSPG on the FASTlab™ (Edwards et al. 2021) were readily adapted and updated to our AIO cassette, except for the reactor emptying step. Subsequent to radiofluorination of the naphthyltosylate precursor (**1**), the protected [^18^F]FSPG intermediate (**[**^**18**^**F]2**, Fig. 1) was deprotected firstly with H_2_SO_4_ (1 M) followed by NaOH (4 M), resulting in a viscous crude mixture. During our initial automation attempts, this reaction mixture was not consistently removed from the reactor for subsequent quenching. This is likely due to either the low diameter outlet tubing of the Trasis reactor compared to the wider-bore tubing employed by the FASTlab™ reactor, or the use of pinch valves to seal the reactor on the Trasis AIO™. Additional reactor pressurisation steps and an extended time to remove the crude to syringe 3 (Fig. 2) did not solve the problem. Finally, keeping the right-sided pinch valve open after adding sodium hydroxide allowed the process to proceed efficiently and reliably.

Isolation and reformulation of [^18^F]FSPG was achieved by adapting the well-established SPE cartridge-based method (Koglin et al. 2011; McCormick et al. 2019; Edwards et al. 2021). Initially, MCX cartridges were employed to trap [^18^F]FSPG in its protonated form, removing it from the crude eluent, whilst other impurities such as [^18^F]fluoride passed through to waste. Elution of [^18^F]FSPG from the MCX cartridges by PBS was facilitated by a pH-driven change in its protonation state. The PBS eluent subsequently eluted the negatively charged [^18^F]FSPG, whilst the lipophilic component of the MCX stationary phase retained any unreacted precursor. [^18^F]FSPG was then eluted through an Alumina N cartridge and a Hypercarb cartridge to remove any residual impurities. Finally, elution through a sterile filter provided the formulated [^18^F]FSPG in excellent RCP and A_m_, without any requirement for HPLC purification.

Whilst the SPE purification above worked well for our initial experiments with lower activities, when higher activities were employed, the radiochemical purity of the formulated [^18^F]FSPG dropped (Fig. 5). This may result from increased radiolysis occurring during the trapping of the [^18^F]FSPG on the MCX cartridges, at which point it is highly concentrated. In addition to a reduction in RCP, radiolysis was likely responsible for the drop in RCY as starting activity increased (Table 2). Importantly, the [^18^F]fluoride impurity could be removed by replacing Alumina N Plus Light and Hypercarb™ cartridges with commercially available Alumina N plus Long cartridges and ENVI-Carb cartridges, respectively. Addition of radioprotectant excipients to the PBS elution buffer, such as ascorbic acid and ethanol, needs further investigation to establish whether this may improve overall RCY. Notably, additional precursor was not required to improve the RCP of the final product, as has been reported for other high-activity productions (Lin et al. 2023), providing substantial cost savings whilst also limiting the risk of chemical impurities. Possible cold impurities generated through precursor degradation during labelling include the corresponding alcohol (substitution of the sulfonate leaving group with water) or alkene (via elimination of the sulfonate). Whilst generation of these compounds in very low amounts is unlikely to have associated toxicity, the structural similarity of these compounds to [^18^F]FSPG may result in affinity for system x_c_^−^, influencing the effective molar activity.

The optimized automated synthesis provided formulated [^18^F]FSPG in just 35 min. To our knowledge, this is the fastest automated production of [^18^F]FSPG to date. The process also represents the first high-activity, cassette-based production of [^18^F]FSPG that doesn’t require bespoke cartridges for purification or external reagents to the cassette/module. Together with the automated cartridge conditioning and precursor solubilisation, the cGMP design will facilitate adoption of the process at other sites and expedite regulatory approval for clinical production.

## Conclusions

We have developed the first automated production of [^18^F]FSPG on the Trasis AIO™. The sequence reliably produced [^18^F]FSPG in amounts suitable for multiple patient doses with high RCY (25.3± 1.1%), RCP (96.8± 1.1%) and A_m_ (372± 65 GBq/µmol). We anticipate that by expanding [^18^F]FSPG automation to the widely used Trasis AIO™, the radiotracer’s availability will substantially increase. Furthermore, we expect the reported purification optimization of high-activity [^18^F]FSPG to be adapted to other modules, expediting the ongoing translation of this important radiotracer at sites requiring clinical production.

## Abbreviations

[^18^F]FSPG: (*S*)-4-(3-^18^F-Fluoropropyl)-L-glutamic acid
PET: Positron emission tomography
[^18^F]FDG: [^18^F]2-fluoro-2-deoxy-d-glucose
GSH: Glutathione
AIO™: AllInOne™
SPE: Solid phase extraction
MCX: Mixed cation exchange
PBS: Phosphate buffered saline
OPA: *o*-Phthalaldehyde
WFI: Water for injection
RCP: Radiochemical purity
A_m_: Molar activity
RCY: Radiochemical yield

## Supplementary Material

Supplementary data

Synthesis sequence

## Figures and Tables

**Table 1:** Cassette positions for reagents and consumables

Valve position (VP)	Reagents, tubing & consumables
01	Short tubing to ^18^O water recovery vial
02	Kryptofix solution (450 μL, A*)
03	Syringe 1 (3 mL syringe)
04	Short tubing to QMA cartridge at VP 05
05	QMA carbonate cartridge (46 mg sorbent)
06	Syringe 2 (Activity Plunger)
07	Short tubing to the 6 mL reactor
08	FSPG precursor vial (**B**)
09	Syringe 3 (20 mL BD syringe)
10	Anhydrous acetonitrile (1.4 mL), 4 mL vial (**C**)
11	Sodium hydroxide (1.9 mL, 4 M), 4 mL vial (**D**)
12	Sulfuric acid (1 M, 7.0 mL), 50 mL vial (**E**)
13	2 ×Oasis MCX Cartridges
14	Syringe 5, 20 mL BD syringe
15	Tubing to sterile WFI bag (100 mL) (**F**)
16	PBS (30 mL), 50 mL vial (**G**)
17	Long tubing to MCX cartridges at VP13
18	Alumina N Plus Long cartridge
Right terminal	Short silicon tubing to right exhaust (waste)
Left terminal	Short silicon tubing to N_2_ gas inlet equipped with 0.2 μm filter
Left exhaust	Tubing to 6 mL reactor

WFI = Water for injection

* Letters refer to reagent positions depicted in Fig. 2

**Table 2:** Synthesis optimisation of [^18^F]FSPG on the Trasis AIO™

Entry	Starting activity (GBq)	Product activity (GBq)	Final SPE cartridge	RCP (%)	RCY (%)
1^a,b^	< 5 GBq	–	Hyper-Carb (500 mg)	96.9 **±** 0.7 (n = 6)	34.6 **±** 6.1 (n = 6)
2^b^	45–75	–	Hyper-Carb (500 mg)	94.0 **±** 1.4 (n = 5)	26.5 **±** 4.4 (n = 5)
3^b^	91–101	–	Hyper-Carb (500 mg)	93.1 **±** 1.7 (n = 4)	22.1 **±** 0.6 (n = 4)
4	45–46	–	ENVI-Carb (2.0 g)	97.0 **±** 0.1 (n = 2)	9.4 **±** 3.4 (n = 2)
5	37–67	7.6–12.5	ENVI-Carb (1.0 g)	96.9 **±** 0.8 (n = 4)	26.2 **±** 0.8 (n = 5)
6	96–124	20–23	ENVI-Carb (1.0 g)	96.8 **±** 1.1 (n = 5)	25.3 **±** 1.1 (n = 5)
7	18–25	4.6–7.6	ENVI-Carb (1.0 g)	97.1 **±** 0.4 (n = 4)	33.5 **±** 4.9 (n = 5)

a Optimisation runs included minor adjustments to sequence.

b Employed Alumina N Plus Light cartridge

## Data Availability

The automated synthesis sequence is provided as Supplementary Material. All other data will be made available on request.
