# Supplementary material for: Compact and cGMP-compliant automated synthesis of [18F]FSPG on the Trasis AllinOne™
Source: EJNMMI Radiopharm Chem. Author manuscript; Available in PMC 2025 Feb 21. (PMC11748660; doi:10.1186/s41181-024-00322-7)
Supplement: Supplementary data [file EMS202788-supplement-Supplementary_data.docx]

**Compact and cGMP Compliant Automated Synthesis of [^18^F]FSPG on the Trasis AllinOne^TM^**

**SUPPLEMENTAL DATA**


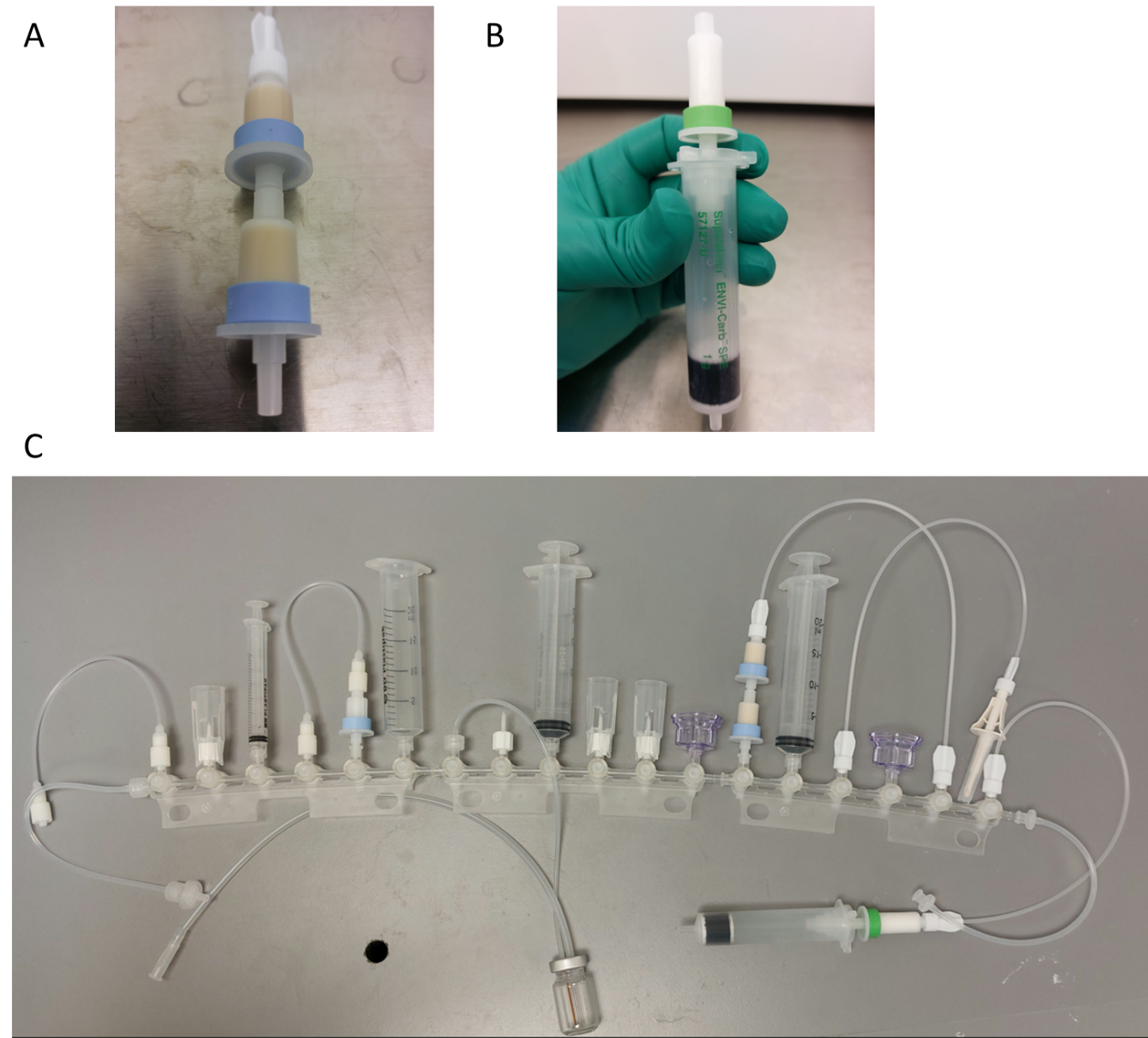


**Supplementary Fig. S1** Cassette Assembly. A) Assembly of MCX cartridges. B) Assembly of Alumina N Long Cartridge and ENVI-Carb cartridge. C) Photo of the cassette before mounting.


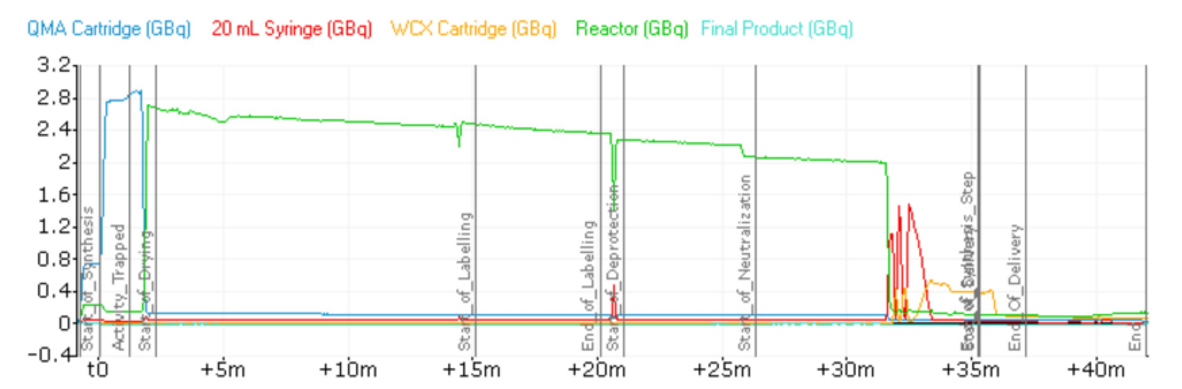


**Supplementary Fig. S2** Synthesis trending example.


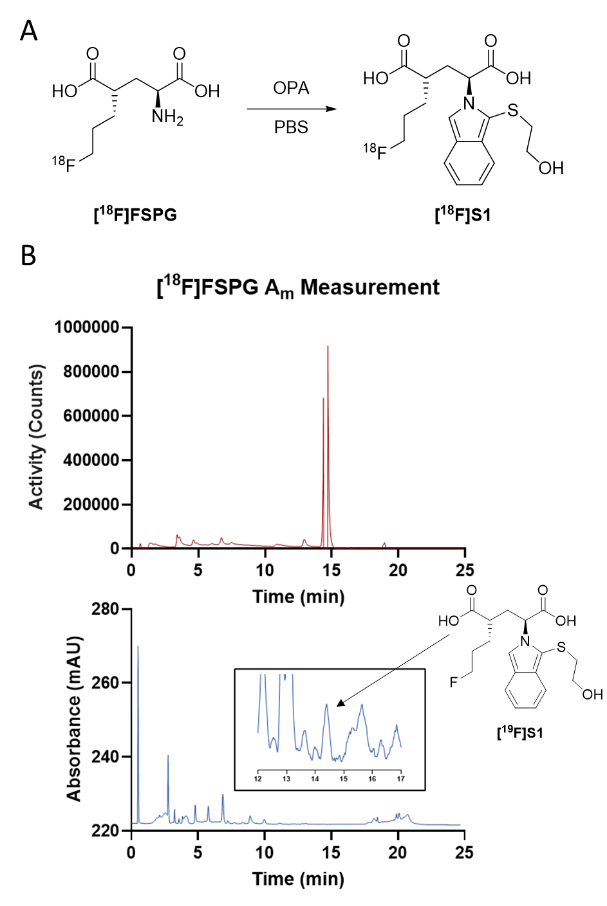


**Supplementary Fig. S3** Measuring [^18^F]FSPG molar Activity. A) Reaction of [^18^F]FSPG with OPA reagent. B) Typical radio and UV chromatograms used to calculate [^18^F]FSPG A_m_. The higher amount of activity required to be injected to measure the A_m_ results in a U-shaped activity peak, consistent with detector saturation.

**Supplementary Fig. S4** Calibration curve for FSPG-OPA adduct.

**
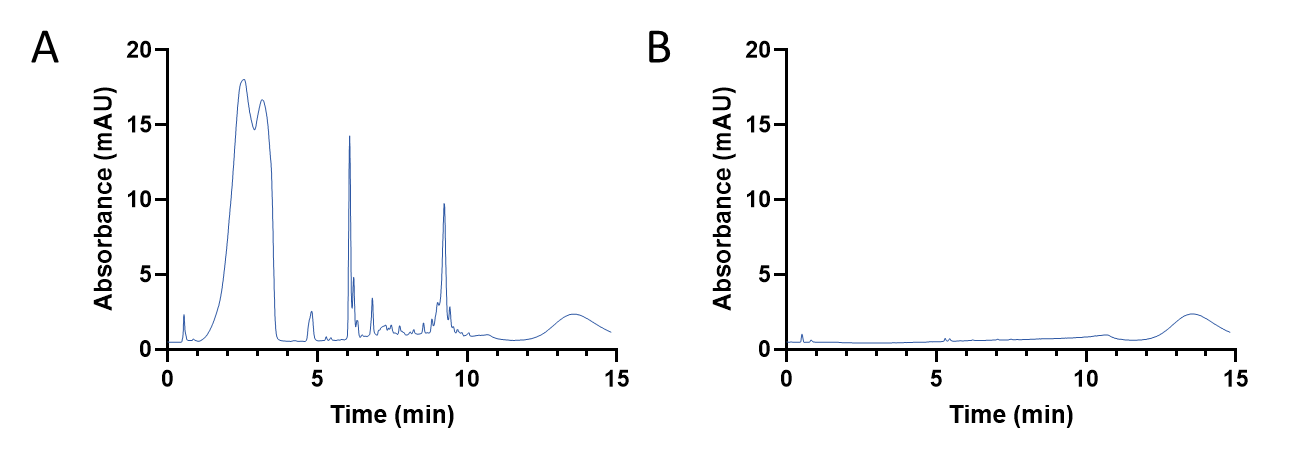
**

**Supplementary Fig. S5** UV chromatograms of neat OPA (A) and PBS formulation buffer (B).

**Supplementary Table S1**

| **Entry** | **Starting**  **Activity (GBq)** |  | **Product**  **Activity (GBq)** | **pH** | **RCP (%)** | **RCY (%)** |
| --- | --- | --- | --- | --- | --- | --- |
| 1 | 18 |  | 4.6 | 7.0 | 96.7 | 33.95 |
| 2 | 24.5 |  | 5.8 | 7.0 | # | 30.45 |
| 3 | 25 |  | 5.2 | 7.0 | 97.1 | 26.77 |
| 4 | 25.3 |  | 7.5 | 7.0 | 97.6 | 38.01 |
| 5 | 25.7 |  | 7.6 | 7.0 | 97.1 | 38.08 |
| 6 | 37 |  | 7.6 | 7.0 | # | 26.44 |
| 7 | 38.8 |  | 8.0 | 7.0 | 97.6 | 26.54 |
| 8 | 39.2 |  | 8.1 | 7.0 | 96.4 | 26.6 |
| 9 | 48.4 |  | 10 | 7.0 | 96.0 | 26.6 |
| 10 | 67 |  | 12.5 | 7.0 | 97.5 | 24.78 |
| 11 | 96 |  | 20 | 7.0 | 95 | 26.81 |
| 12 | 98.6 |  | 20.1 | 7.0 | 97.4 | 26.24 |
| 13 | 113 |  | 21 | 7.0 | 97.4 | 24.69 |
| 14 | 120 |  | 22 | 7.0 | 96.8 | 24.35 |
| 15 | 124 |  | 23 | 7.0 | 97.6 | 24.64 |

# Not recorded due to HPLC equipment malfunction.
